# 

**Published:** 1877-04

**Authors:** 


CONTENTS.

PAGE.

**CONTRIBUTIONS.**

Keeping up Appearances. BEN. H. PRATT............225
School-Life Origin of Nervous Diseases. P. H. HAYES, M. D............227
Probably and Perhaps. THOS. K. BEECHER............228

**MEDICAL ITEMS AND NEWS.**

Sedative Pills for Dry Cough--Hæmoptysis--Hoarseness—Epilepsy—Ergot for Injection—Priapism—Large doses of Ergot—Bronchial Catarrh—Kava-Kava for Gonorrhœa—Incontinence of Urine--Galactagogue—Urticaria--Jaborandi--Blisters--Fetid Feet—Prickley Heat—Chilblains—Carbolized Bran in Compound Fractures—Erysipelas—Leucocythæmia—Syrup of Licorice Root—Bromide of Potassium as a Caustic—Chloroform—Gout and Neuralgia--Puberculosis—Depilatory—Cold in Head—Cream of Camphor—Cough Mixture—Typhoid Fever—Disinfectant—Acute Rheumatism—Acne—Action of Chloral on Rectum—Asthma—Dropsy—Expectoration of Phthisis—Diptheria—Phthisical Laryngitis--Tubercular Consumption—Aspiratory Puncture of Bladder—Secret Pile Remedy—Malarial Chill............230 to 238

**OUR CORRESPONDENCE.**

Seven Letters with Replies............238

**HOUSEHOLD HINTS AND RECIPES.**

Milk—Plastic Material—Hot-Water-Proof Cement—Cement for Tin and Metalic Substances—To clean Globes—Ink for Marking—Liquid Shoe Polish—Chromatized Gelatine as a Cement—Grease Spots on Marble—Restoring Faded Ink—Milk on Carpets—To mend Coal Stoves—Bedbugs—Red Wash for Bricks--Liquid Slating for Black-boards—Ivory Balls Color—Rice, to Cook—In Case of Fire—Treatment of Wine—Walks—Spots on Silk—Soap-stone Hearths, to clean—Candle-Grease, to remove—Spots on Paint and Varnish, to remove—Tin Ware, to clean—Fruit Stains, to remove—Corns—Chromos, to clean—Mirrors, to clean—Tea Leaves, uses—White Linen, to clean--Washing Dishes--Mildew--Eider Down 240 to 245

**EDITORIAL.**

Paralysis of Children............245
Asthenopia............246
The Trials of a Dyspeptic............247
Pets............248
Hard to Please............248

**EDITORIAL CHIT-CHAT.**

Æsthetic—A New Cover—Flower Time—H.—Art Parlors—Late—Time Absorbent—Bile Time—Horseback Riding—Spring Styles—Strong, Story from H.—Weathery—Fond of it—The Telephone—Medical Bill of Fare—Fritz' Sayings—Blue Glass Mania—Conservatory Labors—Temperance Movement............249 to 252

